# Tissue engineered endometrial barrier exposed to peristaltic flow shear stresses

**DOI:** 10.1063/5.0001994

**Published:** 2020-06-02

**Authors:** David Elad, Uri Zaretsky, Tatyana Kuperman, Mark Gavriel, Mian Long, Ariel Jaffa, Dan Grisaru

**Affiliations:** 1 Department of Biomedical Engineering, Faculty of Engineering, Tel-Aviv University, Tel-Aviv 69978, Israel; 2 Center of Biomechanics and Bioengineering and Beijing Key Laboratory of Engineered Construction and Mechanobiology, Institute of Mechanics, Chinese Academy of Sciences, Beijing 100190, China; 3 Department of Obstetrics and Gynecology, Lis Maternity Hospital, Tel-Aviv Medical Center, Tel-Aviv 64239, Israel; 4 Sackler Faculty of Medicine, Tel Aviv University, Tel Aviv 69978, Israel; 5 Gynecological Oncology Unit, Lis Maternity Hospital, Tel-Aviv Medical Center, Tel-Aviv 64239, Israel

## Abstract

Cyclic myometrial contractions of the non-pregnant uterus induce intra-uterine peristaltic flows, which have important roles in transport of sperm and embryos during early stages of reproduction. Hyperperistalsis in young females may lead to migration of endometrial cells and development of adenomyosis or endometriosis. We conducted an *in vitro* study of the biological response of a tissue engineered endometrial barrier exposed to peristaltic wall shear stresses (PWSSs). The endometrial barrier model was co-cultured of endometrial epithelial cells on top of myometrial smooth muscle cells (MSMCs) in custom-designed wells that can be disassembled for mechanobiology experiments. A new experimental setup was developed for exposing the uterine wall *in vitro* model to PWSSs that mimic the *in vivo* intra-uterine environment. Peristaltic flow was induced by moving a belt with bulges to deform the elastic cover of a fluid filled chamber that held the uterine wall model at the bottom. The *in vitro* biological model was exposed to peristaltic flows for 60 and 120 min and then stained for immunofluorescence studies of alternations in the cytoskeleton. Quantification of the F-actin mass in both layers revealed a significant increase with the length of exposure to PWSSs. Moreover, the inner layer of MSMCs that were not in direct contact with the fluid also responded with an increase in the F-actin mass. This new experimental approach can be expanded to *in vitro* studies of multiple structural changes and genetic expressions, while the tissue engineered uterine wall models are tested under conditions that mimic the *in vivo* physiological environment.

## INTRODUCTION

Uterine peristalsis is the accepted terminology for the coordinated spontaneous contractions of the non-pregnant uterus (Myers and Elad, 2017). These contractions have essential roles in human early life (Elad *et al.*, 2020). First, they rapidly transport the sperm through the female genital tract for fertilization of the oocyte in the fallopian tube and then transport the blastocyst to a proper implantation site in the uterine wall during the window of implantation when the uterus is receptive. In addition, during the monthly cycle, these contractions remove menstrual debris and also enable birth of the fetus during labor (i.e., parturition).

The non-pregnant uterus is a peer-like organ with thick walls that have a structural architecture designed to perform its dynamic roles at the onset of human reproduction. The uterine wall is made of three layers. The endometrium is the passive inner layer and made of endometrial epithelial cells (EECs) and endometrial stromal cells (ESCs). The myometrium is the thick active middle layer and made of myometrial smooth muscle cells (MSMCs) organized in fascicles (i.e., fiber-like) in a complex helical architecture (Young and Hession, 1999). The perimetrium is the thin outer layer of connective tissue that merges with the broad ligament. At the endometrial–myometrial interface (EMI), which is also known as the uterine junctional zone, the endometrial cells and glands are in direct contact with the MSMCs without a separating basement membrane (Fusi *et al.*, 2006). At this interface, myometrial contractions deform the endometrial layer and induce peristaltic intra-uterine fluid flow, which is the physical outcome of uterine peristalsis (Eytan and Elad, 1999; Kunz and Leyendecker, 2002; Gora *et al.*, 2018). This region is also thought to be the location of development of uterine diseases such as adenomyosis, endometriosis, or leiomyomata (Brosens *et al.*, 1995; Leyendecker and Wildt, 2011; Shaked *et al.*, 2015; Jorge *et al.*, 2014).

Uterine peristalsis is well accepted as the leading mechanism for the intra-uterine transport of sperm and embryo, which are required for the establishment of early human life via successful implantation of the blastocyst in the uterine wall and beginning of gestation (Ivell, 2017). Details on the biomechanical characteristics of *in vivo* uterine peristalsis can be found in review articles (Myers and Elad, 2017; Chen *et al.*, 2013). The direction of the peristalsis waves is fundus-to-cervix during the menstruation days and at rates of 0.5–2.5 contraction/min. During the proliferative and secretory phases, the waves reverse the direction to cervix-to-fundus at rates of 1–5 contraction/min. Computational modeling of the intra-uterine fluid flow pattern in a 2D model of the closed uterus revealed a maximal velocity of about 5 mm/s, which may be induced on the EECs peristaltic wall shear stresses (PWSSs) of the order of 20 mPa (Yaniv *et al.*, 2009).

Cellular mechanotransduction or mechanobiology refers to cellular mechanisms that convert mechanical stresses from the physical environment to biochemical signals that modify the cellular cytoskeleton response (Ingber, 2006; Wang *et al.*, 2014; Wang, 2017). It has been comprehensively studied in vascular endothelial cells, stem cells, nasal epithelial cells, respiratory epithelium, and musculoskeletal tissues (Jorge *et al.*, 2014; Even-Tzur Davidovitch *et al.*, 2011; Leiphart *et al.*, 2019). Mechanobiology of reproductive tissue is an emergent topic that has been recently discussed in reviews (Elad *et al.*, 2020) but very little if at all has been done. The published *in vitro* models of reproductive tissue have not yet reached the stage, which allow for mechanobiology investigations (Atala, 2012; Hellström *et al.*, 2017).

In this study, we developed a new laboratory setup for application of PWSSs on an *in vitro* tissue engineered model of the endometrial barrier. We implemented the *in vitro* co-culture model of the endometrial barrier that mimics the two-layer anatomical architecture of EECs and MSMCs (Kuperman *et al.*, 2020). The barrier model was co-cultured on new custom designed wells that can be disassembled to allow installation of the biological model in a special chamber for simulations of peristaltic flows. The peristaltic flow patterns were generated by a custom printed belt mechanism that induced time-depended motility of an elastic cover of the medium above the cultured endometrial barrier. We demonstrated that shear stresses much smaller than those recorded in vascular vessels induce alterations in the cytoskeletal actin filaments of both layers of EECs and MSMCs. It is the first evidence that mechanobiology and environmental forces play important roles in modification of the uterine wall ultrastructure.

## RESULTS

**FIG. 1.**
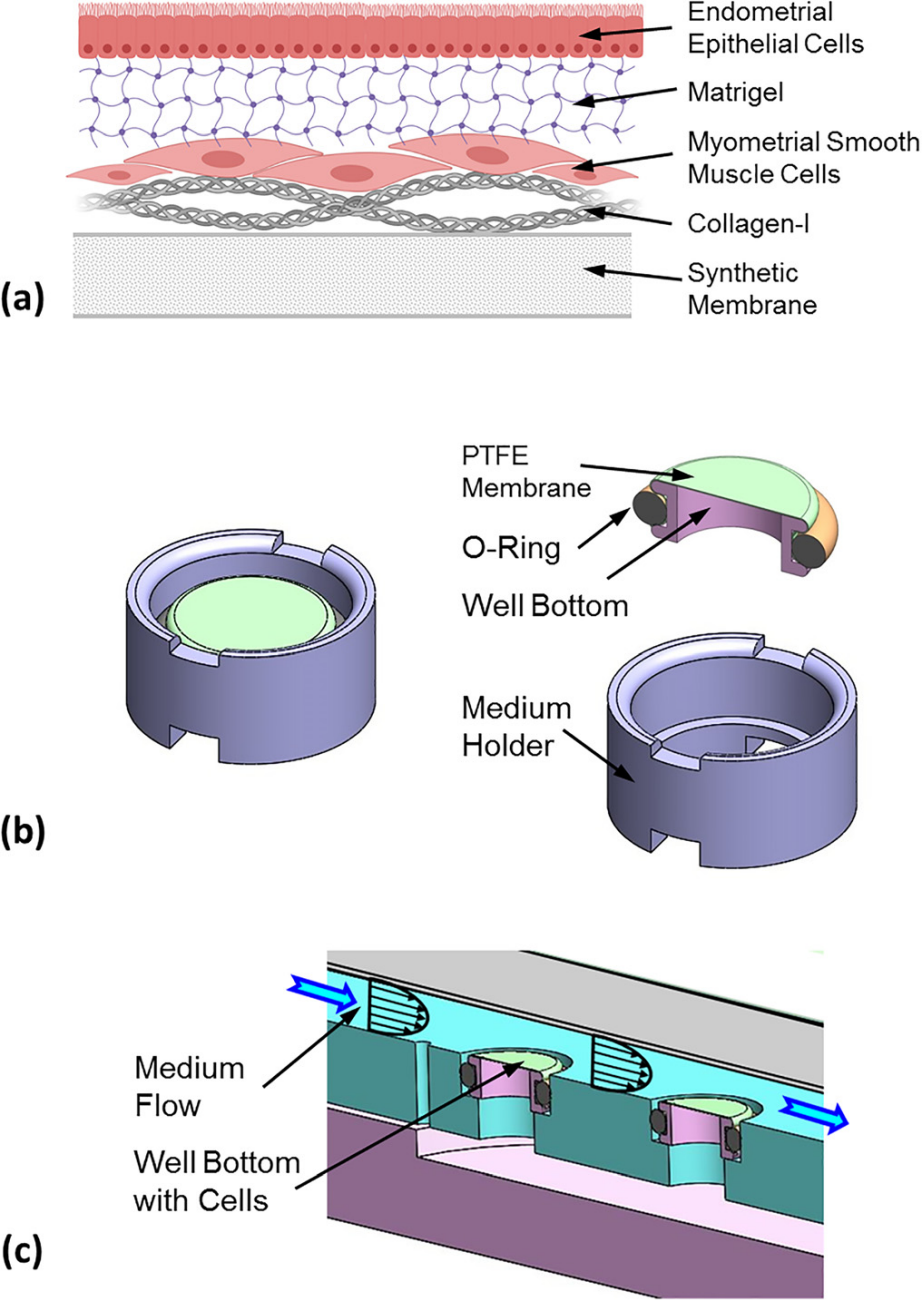
(a) Scheme of the tissue engineered endometrial barrier model. Reprinted with permission from Kuperman *et al.*, Biomech. Model Mechanobiol. (published online, 2020). Copyright 2020 Springer. (b) Custom-designed well with a net diameter of 8 mm for cell culture that can be disassembled for mechanobiology experiments. (c) Example of installment of the well bottom with an *in vitro* biological model in a flow chamber.

The two-layer endometrial barrier model is schematically described in Fig. 1(a). The 3D culture of EECs on top of MSMCs (Kuperman *et al.*, 2020) was successfully reproduced on new and smaller custom-designed wells. This new well was composed of a well bottom with a synthetic membrane and a cylindrical medium holder [Fig. 1(b)]. The model was cultured on the well bottom, which can be disassembled and installed in a test chamber where the cellular model can be exposed to physical environments similar to those existing *in vivo*, for example, a flow chamber [Fig. 1(c)]. The two-layer cellular model was co-cultured on a net area of 50 mm^2^ of a collagen-I coated polytetrafluoroethylene (PTFE) membrane stretched over the well bottom. First, human MSMCs were cultured on the collagen coated PTFE membrane, and 24 h later, human EECs were cultured on the MSMCs after they were coated with a Matrigel layer to simulate the barrier of stromal tissue. On Day, 5 the *in vitro* model was ready for examination and mechanobiology experiments.

**FIG. 2.**
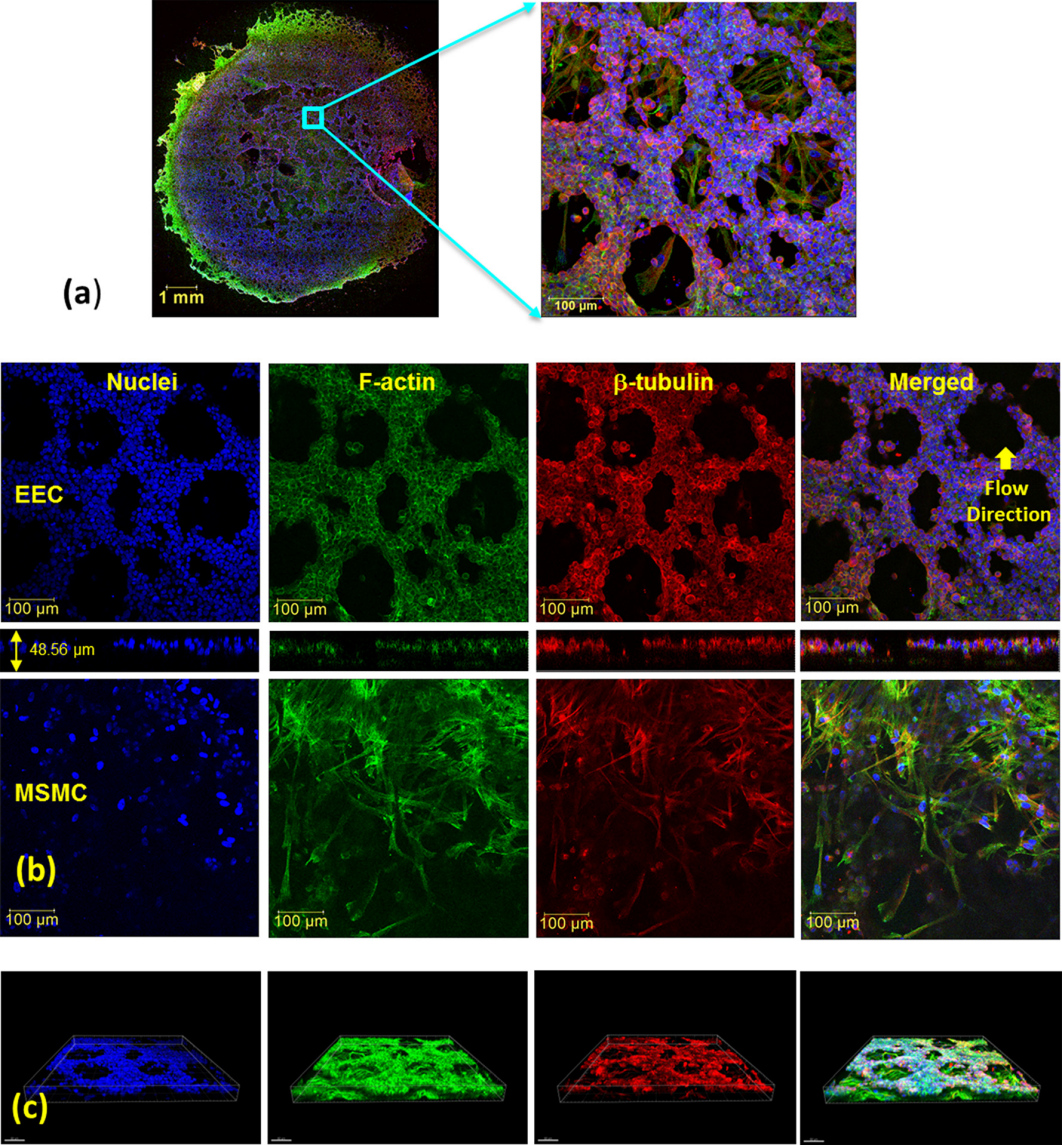
Confocal microscopy images of the endometrial barrier model, which is composed of a co-culture of human endometrial epithelial cells (EECs) on top of human myometrial smooth muscle cells (MSMCs): (a) general image of the whole well bottom from which multiple locations of 578 × 578 *μ*m have been analyzed; (b) images of non-specific staining of F-actin, β-tubulin, and the nucleus of both the EECs and MSMCs along with the Z-section; (c) three-dimensional images of the endometrial barrier model. Antibodies: for F-actin, Cytopainter Phalloidin-iFluor 488 Reagent (ab176753), 1:1000 in DPBS 1% BSA; for β-tubulin, recombinant anti-beta tubulin antibody conjugated [EPR16774] (Alexa Fluor® 555) (ab206627) diluted with DPBS 1:500; and for nuclei, DAPI (D9542).

**FIG. 3.**
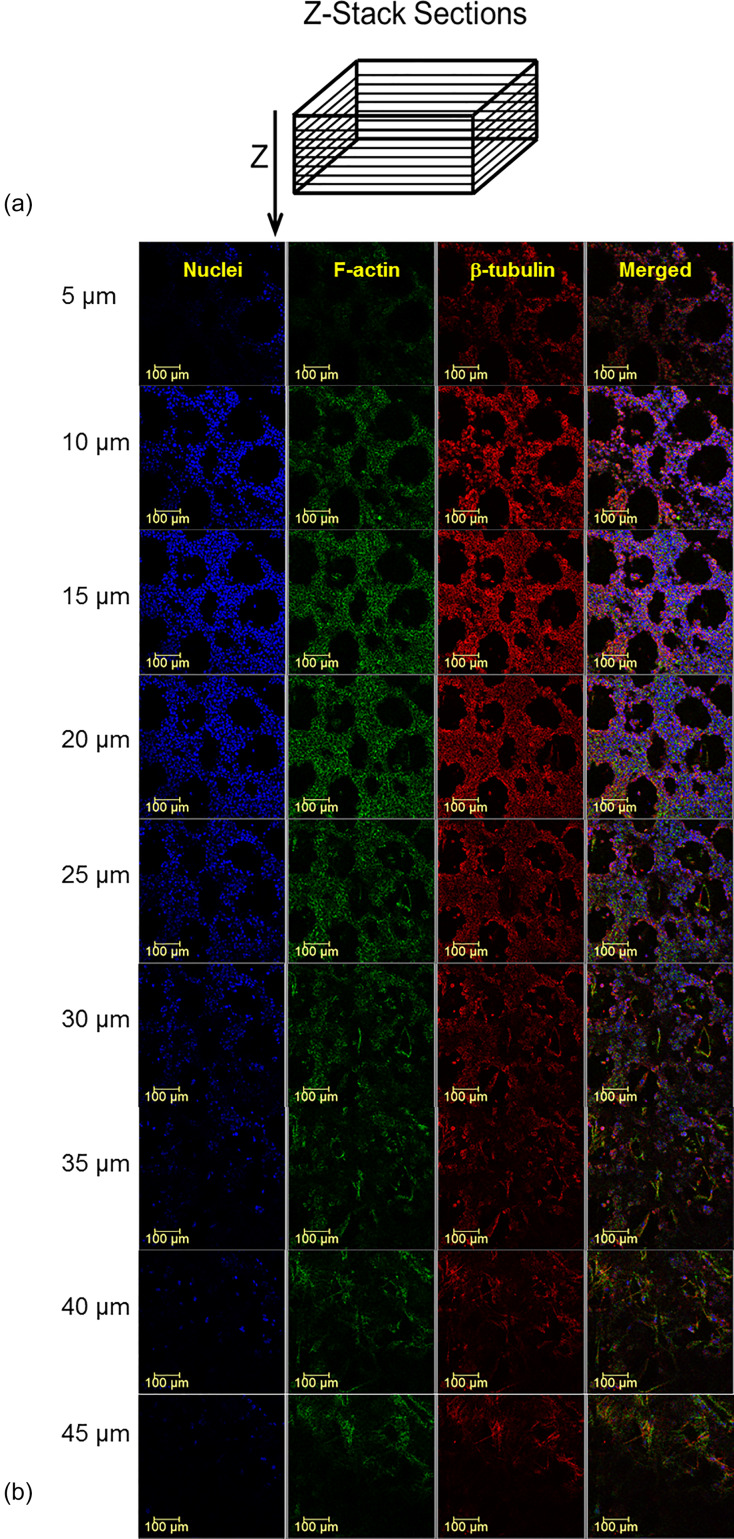
Successive confocal microscopy images of the endometrial barrier model. (a) Scheme of the cross sections perpendicular to the Z-axis. (b) The cross sections at indicated depths starting from the epical end of the endometrial layer. Antibodies: for F-actin, Cytopainter Phalloidin-iFluor 488 Reagent (ab176753), 1:1000 in DPBS 1% BSA; for β-tubulin, recombinant anti-beta tubulin antibody conjugated [EPR16774] (Alexa Fluor® 555) (ab206627) diluted with DPBS 1:500; and for nuclei, DAPI (D9542).

Previously, we proved using larger wells that the endometrial barrier model exhibited the phenotype of human EECs and MSMCs (Kuperman *et al.*, 2020). Here, we used non-specific immunofluorescence markers for confocal imaging of the cytoskeletal components of F-actin, β-tubulin, and the nucleus of both the EECs and MSMCs. The large cultured surface (i.e., 50 mm^2^) was mostly confluent over the well bottom and allowed extraction of multiple locations of 578 × 578 *μ*m [Fig. 2(a)]. The confocal images of the representative cross. section through the EEC and MSMC layers demonstrated that the cultures have reached 90%–100% confluence [Fig. 2(b)]. The EECs also migrated into the Matrigel layer coating and demonstrated the rosette-like shape of the *in vivo* endometrial glandular epithelium (Jin, 2019). Previously, we also demonstrated after hormonal treatment of the endometrium model that the progestogen associated endometrial protein secreted into regions surrounded by but without DAPI staining, which means it resided outside of the EECs (Kuperman *et al.*, 2020). The overall barrier model is a 3D structure as depicted in the 3D plots in Fig. 2(c). Further confirmation for the 3D multi-layer model of the endometrial barrier is provided in Fig. 3, which depicts selected cross sections throughout the transverse Z-stack of the confocal images. It clearly shows that the barrier thickness is 50 ± 20 *μ*m, whereas the EEC layer is approximately 20 ± 10 *μ*m and the MSMC layer is 15 ± 5 *μ*m.

**FIG. 4.**
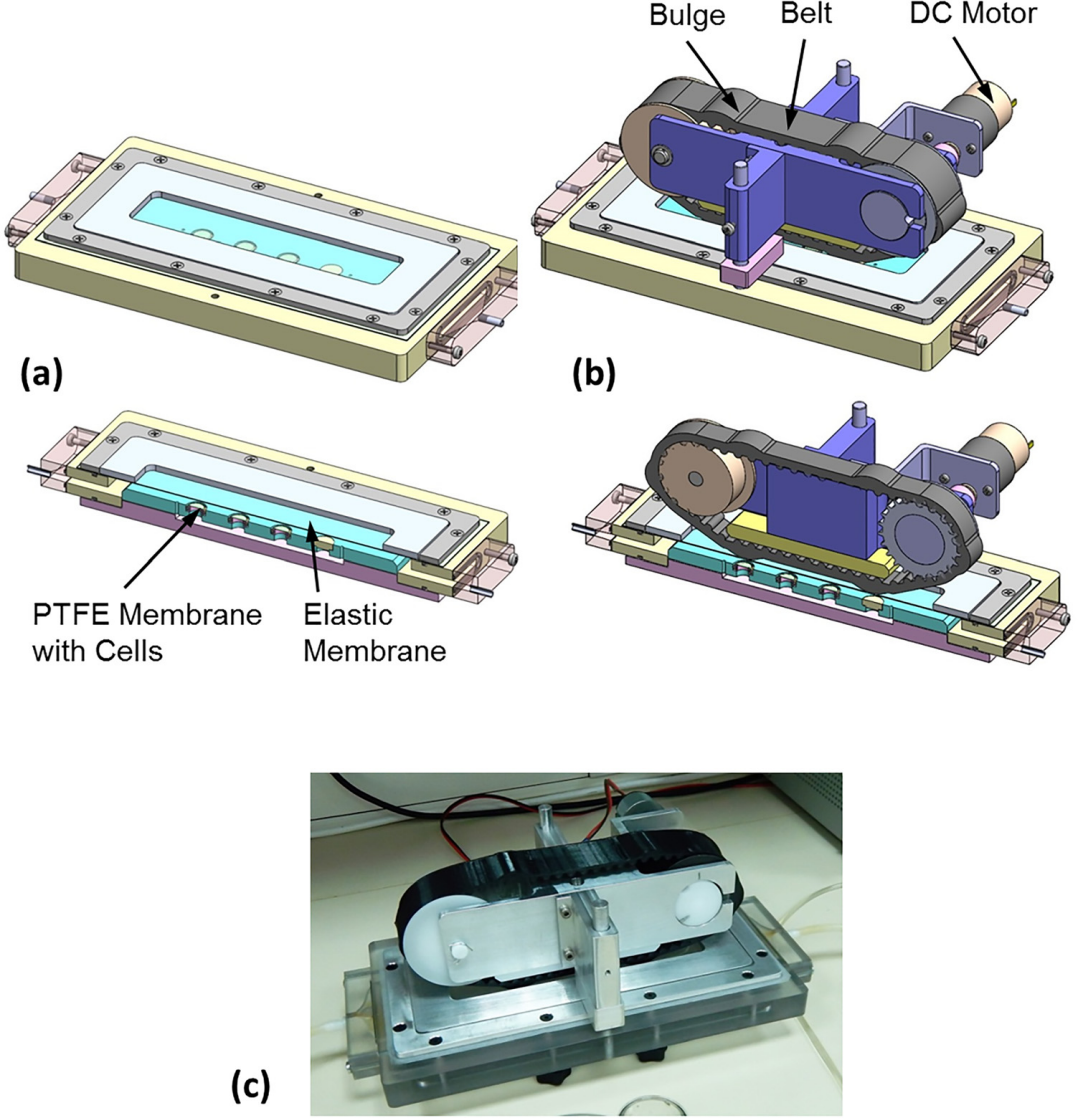
Experimental setup to induce peristaltic flow patterns within a flow chamber. Peristaltic wall shear stresses (PWSSs) are applied on the tissue engineered endometrial barrier model installed at the bottom of the chamber. (a) The flow chamber with the cultured cell at the bottom and a cover of an elastic membrane. (b) The driving mechanism to induce time dependent wall motility of the elastic membrane on top of the flow chamber. (c) Picture of the experimental setup.

The main goal was to apply PWSS on top of the *in vitro* endometrial barrier model. Peristaltic flows can be generated by the propagation of a wall displacement wave along a conduit with flexible walls (for example, Shapiro *et al.*, 1969; Jaffrin and Shapiro, 1971; Eytan and Elad, 1999; Eytan *et al.*, 2001). To establish this concept, we built a rectangular flow chamber with rigid walls except of the upper wall that was made of a flexible membrane to allow for cyclic wall displacements. The chamber's bottom accommodated up to four well bottoms with the endometrial barrier model [Fig. 4(a)]. A closed compartment filled with a culture medium underneath the bottom provided nourishment for the biological models during flow experiments. The flow chamber ends were connected with tubes to a closed flow circuit filled with the culture medium. The cyclic wall motility of the flexible membrane at the top of the flow chamber was introduced via a simple belt transmission mechanism with a custom designed synchronous (i.e., toothed) belt that was printed from a flexible material [Fig. 4(b)]. The belt with curved teeth was stretched between a cylindrical pulley and a matching toothed pulley connected to a DC motor that run at a constant speed [Fig. 4(b)]. The flat outer surface of the belt included periodic parabolic bulges, which were pressed against the elastic membrane in a way that moving the belt induced time-dependent wall motility of the top elastic membrane. This wall motility generated the required peristaltic flow pattern within the flow chamber and on top of the biological model at the bottom of the chamber. The new experimental setup for exposing tissue engineered models to PWSS is shown in Fig. 4(c). More information can be found in Figs. S1–S4 and Videos 1 and 2 in the supplementary material.

**FIG. 5.**
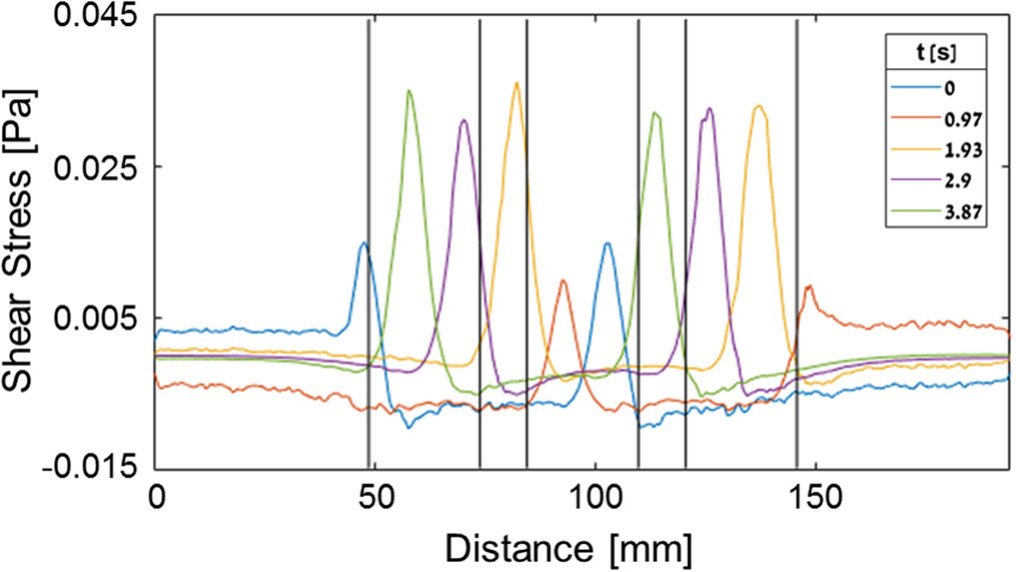
Computed peristaltic wall shear stresses (PWSSs) at the bottom of the flow chamber along the centerline of the co-cultured endometrial barrier model.

To explore the pattern of the PWSSs exerted on the endometrial barrier models, we conducted a computational analysis by exporting the 3D fluid domain within the flow chamber into the finite elements [commercial software of ADINA (Fig. 5)]. We assumed laminar incompressible flow of the culture medium with a density and a viscosity of 1000 kg/m^3^ and 0.001 Pa⋅s, respectively. The fluid volume was meshed by approximately 1 000 000 elements, and the membrane displacement varied with the belt geometry that moved with a linear velocity of 12 mm/s. The resulted time dependent PWSSs on top of the endometrial barrier models are depicted in Fig. 5. The cyclic shear stresses reach values as high as 0.045 Pa, which move along the well bottoms when the peak wall deformation leaves a local minimal gap of only 2 mm above the cultured cells.

The experiments for exposure of the endometrial barrier model to PWSSs were conducted with 12 well bottoms for each test: 4 for control (i.e., no exposure), 4 for 60 min exposure, and 4 for 120 min exposure. Preparation for the exposure tests required installation of four well bottoms in the bottom of the flow chamber and connecting the closed chamber to a peristaltic pump that slowly filled the whole flow circuit with the culture medium. This filling phase was also conducted for the control well bottoms that were not exposed to PWSSs. Then, the medium filled flow circuit was disconnected from the pump, and the driving mechanism was adjusted on top of the flow chamber in a way that the peaks of the belt bulges locally indented the elastic membrane 3 mm downward leaving a minimal local fluid gap of 2 mm. We conducted two sets of experiments, whereas in each, we exposed four well bottoms with the endometrial barrier model to PWSSs for 0 (i.e., control), 60, and 120 min.

**FIG. 6.**
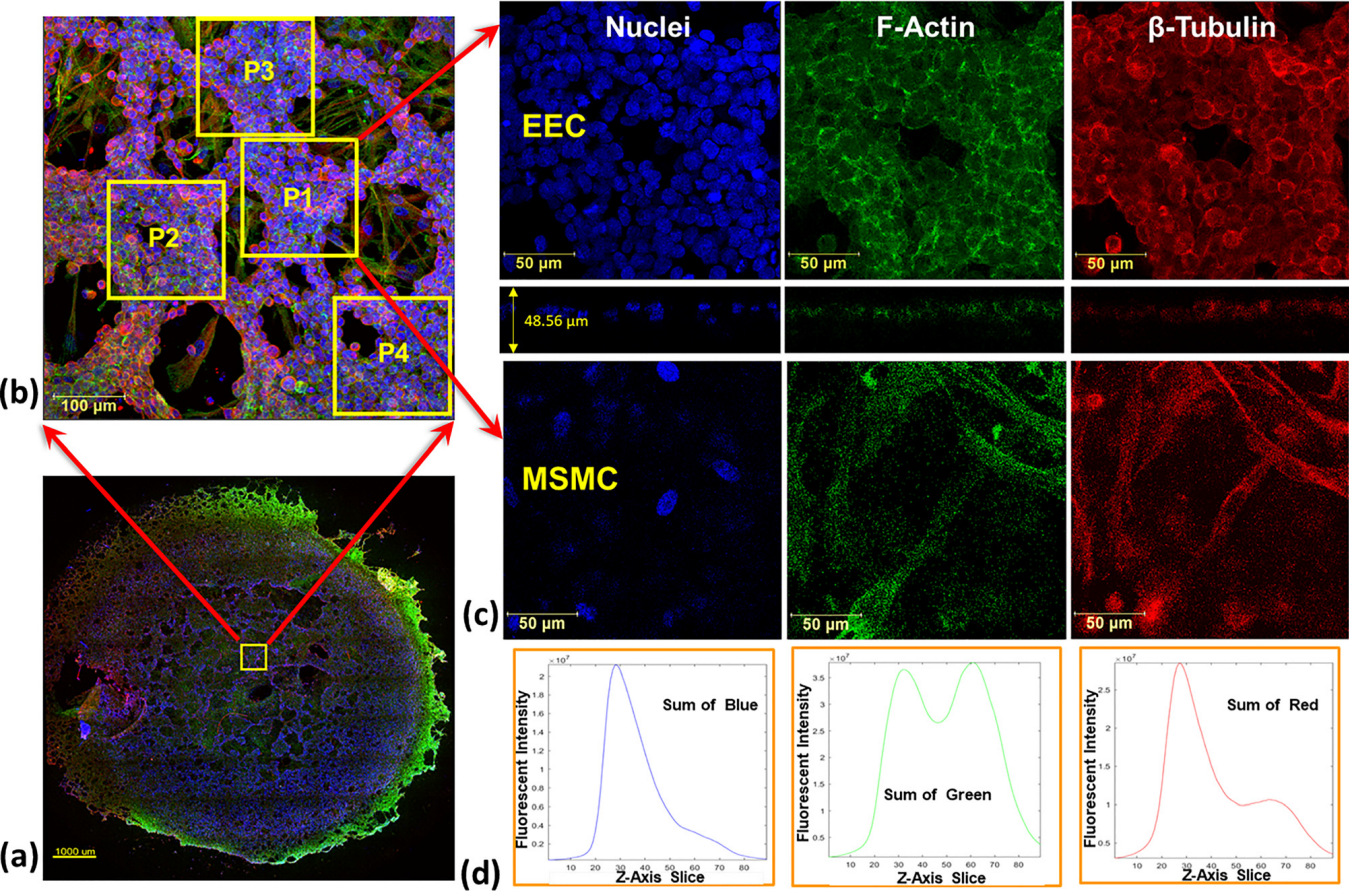
Analysis of cytoskeletal alterations from confocal microscopy images of the endometrial epithelial cells (EECs) co-cultured on top of the myometrial smooth muscle cells (MSMCs). (a) Several locations of 578 × 578 *μ*m were selected from the well. (b) From each of the samples [as shown in (a)], several smaller sub-samples of 200 × 200 *μ*m were used to analyze the mass of cytoskeletal components. (c) Example of the sub-samples of F-actin and β-tubulin images from a control well used for the analysis. (d) Dimensionless sum of the color intensity of cellular components (e.g., nuclei, F-actin, and β-tubulin) from all the cells (i.e., EECs and MSMCs) of the endometrial model.

The cytoskeletal alterations of EECs and MSMCs in response to exposure to PWSSs were quantified from the immunofluorescence staining images of actin and tubulin. Since the fluorescence emission is the outcome of connection between the color marker and the specific molecules, we assumed that the amount of the reflected color intensity of the fluorophore directly correlates with the mass of the marked protein filaments. For this purpose, we zoomed in sample areas of 578 × 578 *μ*m (i.e., 1024 × 1024 pixels) of the confocal images (Fig. 2) and selected regions of smaller sub-samples of 200 × 200 *μ*m (i.e., 352 × 352 pixels) as demonstrated in Fig. 6. A code was developed to sum up the intensity of single colors from all the cross sections of the sub-sample Z-stack as shown in Fig. 6(d). In order to identify the Z-stack cross sections of each layer of the EECs and MSMCs, we conducted a manual search.

**FIG. 7.**
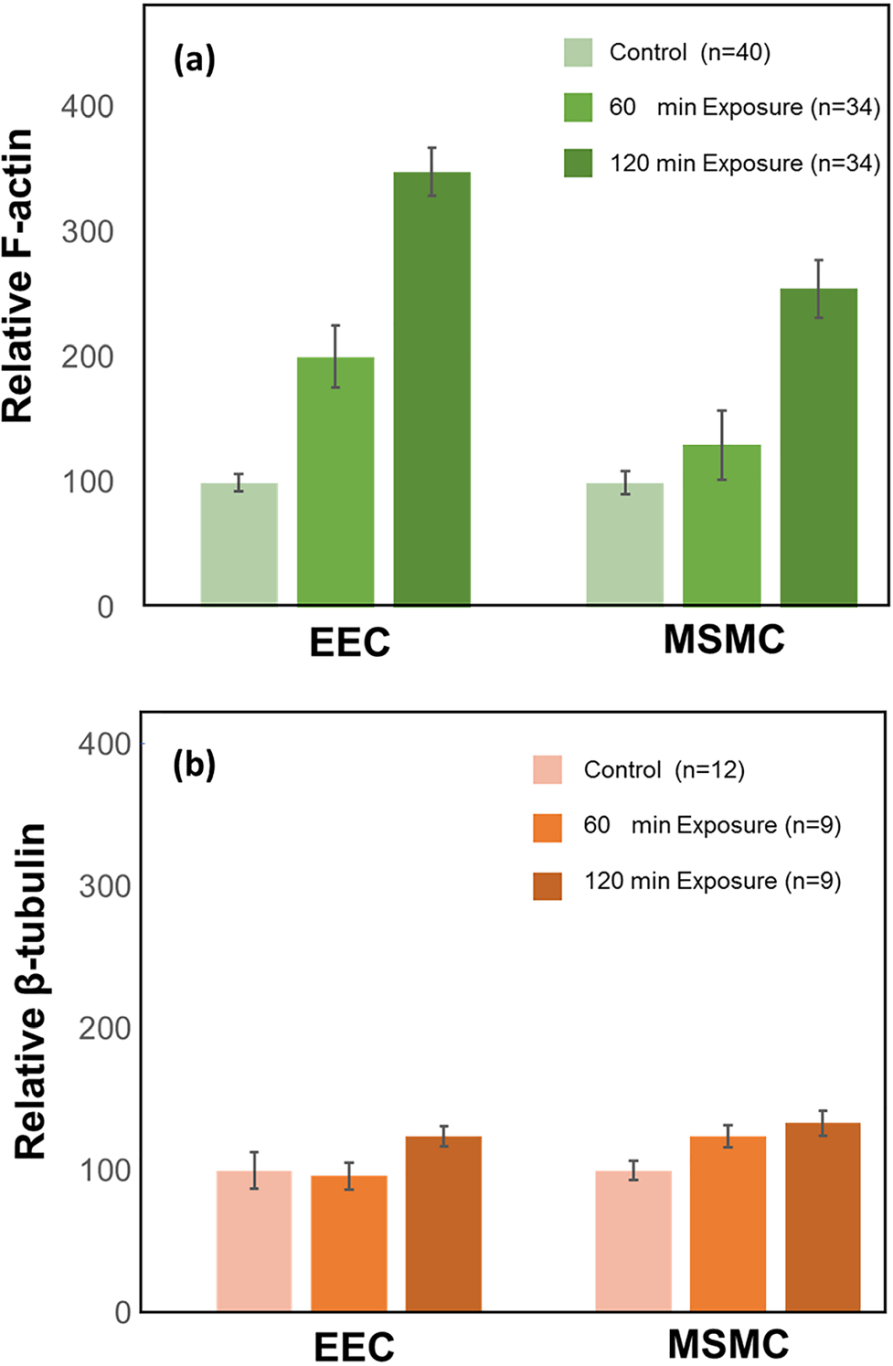
Cytoskeletal alterations of F-actin and β-tubulin in the layers of the endometrial epithelial cells (EECs) and the myometrial smooth muscle cells (MSMCs) after 60 min and 120 min exposure to peristaltic wall shear stresses (PWSSs). The results were computed from multiple sub-samples of a single test with a total of 12 wells. The data after exposure for 60 min and 120 min were scaled by the averaged data for the non-exposed control. (a) Relative amount of F-actin. (b) Relative amount of β-tubulin. The increase in F-actin due to exposure to PWSS is significant (p < 0.05) in both EECs and MSMCs.

**FIG. 8.**
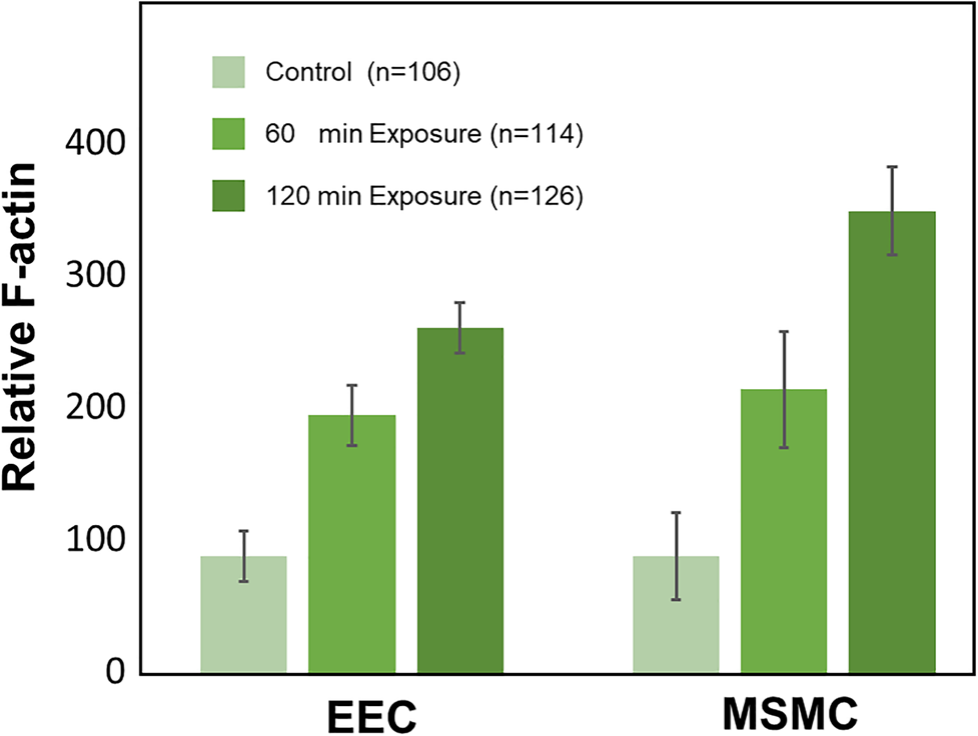
The relative increase in F-actin in response to peristaltic wall shear stresses (PWSSs) of 60 min and 120 min from multiple sub-samples of two sets of experiments with 12 wells in each. The increase in F-actin due to exposure to PWSS is significant (p < 0.05) in both the endometrial epithelial cells (EECs) and the myometrial smooth muscle cells (MSMCs).

The images of a complete set of exposure test (i.e., 12 wells for 0, 60, and 120 min) were acquired at a single session and setting of the confocal microscope. For each exposure, we obtained about 40 sub-samples [as demonstrated in Figs. 6(a) and 6(b)] and calculated the intensity of actin (i.e., green) and tubulin (i.e., red) for each layer of the EECs and MSMCs. The values of intensity from all sub-samples for each exposure (i.e., 60 and 120 min) were averaged and scaled with respect to the average of the non-exposed control (i.e., 0 min). The resulted cytoskeletal alteration for a single test from 12 wells is depicted in Fig. 7 for F-actin and β-tubulin of the EECs and MSMCs. The amount of F-actin filaments increased significantly in response to exposure to PWSSs as compared with the unexposed control for both EECs and MSMCs [Fig. 7(a)]. A longer provocation further increased the amount of F-actin filaments. However, the amount of β-tubulin filaments did not change due to the PWSSs provocation [Fig. 7(b)]. The results for the F-actin relative response to PWSSs shown in Fig. 8 were analyzed from over hundred sub-samples that were extracted from 2 sets of experiments. It clearly demonstrates the significant increase (p < 0.05) in the number of F-actin filaments for increased lengths of exposure to PWSSs.

## DISCUSSION

The response of the endometrial barrier to PWSSs due to uterine peristalsis has been studied for the first time in a laboratory setup. The new engineered endometrial model of co-culture of EECs on MSMCs was implemented in new small custom-designed wells that can be disassembled for mechanobiology experiments. A new setup was developed to induce peristaltic fluid flows in a rectangular flow chamber equipped with the tissue engineered endometrial models at the bottom wall. Exposure of the uterine wall model to PWSSs for 60 and 120 min revealed a significant increase in the polymerization of F-actin filaments in both EECs and MSMCs layers, while the β-tubulin filaments seemed to remain unaltered.

The increase in F-actin polymerization is more pronounced in the EECs, which were in direct contact with the shearing fluid (Fig. 7). Yet, the inner layer of MSMCs also responded to the PWSSs even though it was not in direct contact with the moving fluid. In this study, we utilized the co-culture model of EECs and MSMCs without the ESCs in order to enable the analysis of cytoskeletal changes in each layer of cells separately. The thin Matrigel layer allowed for some migration of EECs, which appeared to be in contact with the MSMCs. Nevertheless, the continuous Matrigel layer transferred physical loads to the lower layer of MSMCs, which accordingly underwent smaller levels of alterations in response to the PWSSs, but yet these alterations are still significant when compared to the unstressed control. It should also be noted that the PTFE membrane most likely underwent small displacements due to the time-dependent PWSSs, which resulted in displacements in the barrier model including the layer of MSMCs.

The cytoskeleton is made of actin filaments, microtubules (e.g., β-tubulin), and intermediate filaments. The exposure of the *in vitro* endometrial barrier to PWSSs revealed a significant increase in the amount of F-actin filaments, while the amount of β-tubulin filaments remained almost unchanged (Fig. 7). It is well established that the polymerization of actin filaments (i.e., stress fibers) is the driving force for cell migration and protrusion (Mogilner and Oster, 1996; 2003; Borisy and Svitkina, 2000; Osborn *et al.*, 2006; Greene *et al.*, 2009; Blanchoin *et al.*, 2014; Schaks *et al.*, 2019). Comprehensive studies also revealed that exposing cells to external mechanical stimulations such as fluid shear stresses lead to increase in actin filaments along with their alignment in certain geometries (Shyy and Chien, 2002; Mengistu *et al.*, 2011; Guolla *et al.*, 2012; Shao *et al.*, 2015). In the present experiments, the cyclic shear stresses traversed the surface of the *in vitro* endometrial barrier with a period of 7.4 s, which may explain the significant response of the actin filaments, which are characterized by fast turnover rates of seconds (Vallotton *et al.*, 2004; Vogel and Sheetz, 2006).

The level of shear stresses in the current study (<0.05 Pa) was much smaller than those known from medium size blood vessels or pulmonary airways (Shav *et al.*, 2014; Green, 2004). However, the present values were of similar order to our predictions in computational models (Yaniv *et al.*, 2009). Regardless of the small shear stresses, we could observe significant alterations in the amount of F-actin filaments after exposing the cellular models to PWSSs for 1 or 2 h (Figs. 7 and 8). Biological environments with similar low levels of shear stresses were also reported in the nasal cavity, intestines, urinary vessels, and liver sinusoids. The maximal computed shear stresses at the air–wall interface within models of the nasal cavity were in the range of 0.2–1.2 Pa (Elad *et al.*, 2006), while *in vitro* exposure of nasal epithelial cells to cyclic stresses up to 0.05 Pa or 0.5 Pa revealed cytoskeletal and functional alterations (Davidovich *et al.*, 2011). Similarly, immortalized human kidney proximal tubule cells exposed to shear stresses of 0.08 Pa responded to cytoskeletal reorganization and functional alterations (Sakolish and Mahler, 2017), and a co-culture model of the liver sinusoidal structure was exposed to shear stresses of up to 0.05 Pa (Du *et al.*, 2017). Microfluidic studies with human intestinal epithelial cells revealed structural and functional changes with shear stresses of up to 0.003 Pa (Delon *et al.*, 2019). Similar shear stresses of 0.05–0.15 Pa also induced cytoskeletal alterations in cultures of epithelial ovarian cancer cells (Avraham-Chakim *et al.* 2013). Currently, it is widely accepted that small environmental stresses have a large impact on the structure and function of biological tissues (Ergir *et al.*, 2018).

A major limitation of this work is the absence of ESCs between the EECs and MSMCs layers of the endometrial model. However, this simplified model of two layers responded to PWSSs with a significant increase in F-actin filaments in both layers. This outcome may support hypotheses that biomechanical signals have some roles in development and performance of the healthy uterus and in etiology of uterine diseases. Another limitation is the short term exposure in the laboratory setup as compared to *in vivo* stresses that exist in the uterus for years. Nevertheless, the relatively fast response of F-actin to mechanical signals allowed us to measure significant changes within 2 h of provocation.

In conclusion, we developed experimental tools for exposing *in vitro* models of the uterine wall to peristaltic flow stresses that mimic the *in vivo* intra-uterine physical environment. Exposure of the tissue engineered model to PWSSs for up to 2 h resulted in significant polymerization of the F-actin filaments in both layers of the EECs and MSMCs. Moreover, the inner layer of MSMCs, which was not in direct contact with the fluid also, responded with increase in the F-actin mass. This experimental approach may be utilized in comprehensive research programs to explore structural changes or molecular expressions of the uterine cellular components due to the uterine physical environment.

## METHODS

### Tissue engineered endometrial barrier

The *in vitro* two-layer endometrial model was co-cultured from commercial EECs on top of MSMCs in new custom-designed wells, as shown schematically in Fig. 1(a), and ethics approval is not required. The new well was composed of a well bottom and a cylindrical medium holder both made of 316 stainless steel and a Viton O-ring [Fig. 1(b)]. A synthetic PTFE porous membrane (Millipore, BGCM00010 Biopore Membrane, 0.4 μm) was mounted on the well bottom to serve as the substrate for the co-culture model. The net diameter for cell culture on the membrane was 8 mm (i.e., cross section of 50.27 mm^2^). Once the tissue engineered model was ready, the well was disassembled for installation of the well bottom with the model in a new flow chamber for investigation of the cellular model response to the peristaltic flow pattern. The concept of this custom-designed well is similar to previous designs we have developed for other mechanobiology studies (Even-Tzur *et al.*, 2006; Levkovitz *et al.*, 2013; Shav *et al.*, 2014).

The endometrial barrier model was similar to the model we have developed on the larger special wells (Kuperman *et al.*, 2020). The human endometrium cell line was used for the EECs (RL95–2, ATCC® CRL-1671™). Human primary uterine smooth muscle cells were used for the MSMCs (ATCC® PCS-460-11™). A vascular cell basal medium was used for the MSMCs (PCS-100-042). For the EECs, we prepared a mixture (1:1) of Ham's F-12 and DMEM (Biological Industries 01–170-1 A) supplemented with 10% bovine serum, 2 mM L-glutamine, 5 mg/ml insulin, and 0.1% penicillin-streptomycin-amphotericin (Pen-Strep-Amp) (Biological Industries 03–033-1B).

The multi-layer model of EECs and MSMCs was co-cultured to confluence of 90%–100% within 5 days from cells of passages 4 through 8 in the humidified incubator at 37 °C and 5% CO_2_. The assembled wells with the PTFE membrane were placed in a 12-well plate. On day 1, the PTFE membrane on the well bottom was coated with 10 *μ*g/cm^2^ bovine collagen Type-I (Sigma C4243) and placed for 24 h in the incubator. On day 2, excess fluid was removed, and the well was washed with Dulbecco's phosphate buffered saline (DPBS) without calcium and magnesium. Then, the moist well was seeded with 75 000 MSMCs (i.e., maximum volume of 100 *μ*l of MSMCs with the medium). On day 3 and after 24 h of culture, the MSMC layer was coated with 10 *μ*l Matrigel (Sigma E1270) diluted 1:1 with DPBS. The well was returned to the incubator for about 1 h for polymerization and stiffening, and then, 150 000 EECs were seeded (i.e., maximum volume of 100 μl of EECs with the medium). On day 4 and after the co-cultured cells grew to 80%–90% confluence, the well was filled with a mixture (1:1) of both mediums for the EECs and MSMCs (i.e., we filled the entire 12-well plate with the wells). On day 5, the co-culture model was ready for the mechanobiology experiments with the new setup for applying PWSSs.

### Experimental setup to induce peristaltic flow

We developed a laboratory experimental setup to induce peristaltic flow patterns within a chamber equipped with the tissue engineered endometrial barrier. The PWSSs applied on the co-culture model mimicked the *in vivo* intra-uterine environment due to nonpregnant uterine peristalsis. The general concept was to have a flow chamber in which one of the fluid boundaries can be deformed in a prescribed time-dependent pattern. Accordingly, the setup was composed of a flow chamber with one flexible wall and a driving mechanism to induce time-dependent wall motility of this flexible wall [Fig. 4(a)]. The fluid-filled rectangular flow chamber (i.e., 337 × 117 × 5 mm) was manufactured from polycarbonate, and the bottom plate can hold four well bottoms with the endometrial barrier model. Underneath the bottom plate, we built a closed compartment filled with the culture medium for nourishment of the biological models during flow experiments. The chamber's upper cover was a silicon membrane of 1.0 mm thickness to allow deformation of the fluid interface. The driving mechanism was based on principles of the simple open belt drive with a custom designed belt with local bulges to induce the prescribed wall motility of the elastic membrane and consequently the adjacent fluid interface.

The custom-designed belt was a synchronous belt of curvilinear teeth with 32 mm width to fit a commercial timing belt pulley (Model 184–331, RS, UK). On the outer diameter, the belt had six symmetrically organized parabolic bulges of 3.6 mm height and 25 mm length, which were spaced about 89 mm one from each other along the circumference [Figs. 4(b) and S4]. The belt was 3D printed from POLYFLEX, which is a semi-elastic material. The belt was stretched between a gear wheel and a smooth idler pulley and was driven using a 12 V DC gear motor (Model 37Dx50L, Hasinen, Taiwan) at a constant speed of 4 rpm. The linear velocity of the bulges above the elastic cover was 12 mm/s. The driving assembly was adjusted over the elastic membrane of the flow chamber via two poles and spacers to allow for the required static maximal indentation of the membrane (e.g., 3 mm in this work). The membrane outer surface was covered by an aluminum plate with a slot of the belt width to allow indentation of the membrane without any deformation beyond the belt width. When the belt was moving the elastic membrane followed by the shape of the belt bulges, it induced time dependent motions at the fluid interface, which, in turn, generated a peristaltic flow pattern within the flow chamber equipped with the endometrial barrier models at the bottom surface. Exploded views of the flow chamber and the driving mechanism are provided in the supplementary material.

In order to support the validity of the new laboratory apparatus to introduce PWSSs on top of the endometrial barrier model, we also conducted a computational model of the fluid domain within the flow chamber while subjected to time-dependent wall displacement as imposed by the moving belt on top of the elastic membrane. Assuming unsteady laminar incompressible water at room temperature (e.g., ρ = 1000 kg/m^3^ and *μ* = 0.001 Pa·s), we received oscillating PWSSs at the bottom of the flow chamber with peak values of up to 0.05 Pa (Fig. 5). The peak values of the PWSSs move at the speed of the belt, while the relative decreases occur due to the recirculation of the fluid that reversed its velocity direction in the wake of the maximal deformation of the elastic wall. The accuracy of the computational results was estimated to be less than 5%. In a previous 2D computational model of uterine peristalsis within a closed uterus model, we received maximal velocities that may result in shear stresses on similar orders (Yaniv *et al.*, 2009).

### Protocol of experiments

On day 5, when the co-cultured endometrial barrier model was ready, we removed the well bottoms from the cylinder holders and installed them into the bottom of the sterilized flow chamber with the EECs facing up the flow. The outlets of the flow chamber were connected with sterile tubes to a small reservoir and a peristaltic pump slowly streamed culture medium into both spaces under and above the well bottoms with the cellular model. The medium was a mixture 50/50 of the medium cultures for EECs and MSMCs that were conditioned in the incubator at 37 °C and 5% CO_2_ for 1 h before filling the system. When the flow chamber was full with the medium without air bubbles, we turned off the peristaltic pump and assembled the driving mechanism on the flow chamber. It was assembled in a way that the bulges of the belt deformed the elastic membrane 3 mm downwards so that the fluid domain above the model will change between 2 and 5 mm while the belt is moving.

Exposure of the endometrial barrier model to PWSSs was done for either 60 min or 120 min, while the setup is within the humidified incubator. The non-exposed control was treated with the 1 h filling procedure within the flow chamber but has not been exposed to PWSSs. We co-cultured a total of 12 wells for each experiment: 4 for control, 4 for 60 min exposure, and 4 for 120 min exposure. We repeated the experiments 3 times. After exposure to PWSSs,, we stained all the well bottoms, including the unstressed controls, for immunofluorescence imaging and quantification of the actin and tubulin filaments of the EECs and MSMCs.

### Immunofluorescence and confocal microscopy

The co-culture of EECs and MSMCs over the PTFE membrane was assessed using standard staining and microscopy protocols. The tissue engineered model was first introduced into ice cold PBS and then fixed in paraformaldehyde 4% for 15 minutes. Then, it was permeabilized in 0.5% Triton X-100 for 15 minutes and blocked in 1% bovine serum albumin (BSA) for 60 minutes. The cytoskeleton structure of both MSMCs and EECs was stained with the same antibodies. For the F-actin filaments, we used Cytopainter Phalloidin-iFluor 488 Reagent (ab176753) 1:1000 in DPBS 1% BSA. For the β-tubulin filaments, we used the recombinant anti-beta tubulin antibody conjugated [EPR16774] (Alexa Fluor® 555) (ab206627) diluted with DPBS 1:500. The nuclei were stained with DAPI (D9542). The cultured cells were examined under a Leica SP8 confocal microscope with dry X20 lens. Analysis and quantification of the images were done using the LAS-X (Leica) and Matlab, and 3D images were analyzed with Imaris.

### Quantification of F-actin and β-tubulin contents from fluorescence imaging

We assumed that the intensity of the reflected color of the antibody directly correlates with the mass of the marked cellular protein. For example, the F-actin filaments were visualized using Phalloidin (i.e., iFluor 488), which appears green in the confocal images. We quantified the intensity of a specific color using the Z-stack, which is composed of 2D images of cross sections throughout the sample thickness [Figs. 2(a) and S5(a)]. In each experiment, we had up to 12 wells for the non-exposed control and after 60 and 120 min exposure to PWSSs. All the confocal images of a single experiment were acquired at a single setting of the microscope.

The wells of the present study have a net cultured surface of 50.27 mm^2^ (i.e., a diameter of 8 mm), which allowed acquisition of multiple samples of 578 × 578 *μ*m (i.e., 1024 × 1024 pixels) from each well [see Fig. 2(a)]. The intensity data of the stained colors of the Z-stack of cross-sectional images were exported by the Leica software LAS X. We converted the original uncompressed LIF file of a single color (i.e., a single channel) into a TIFF grayscale format (i.e., intensity values of green from 0 to 255 for each pixel) to be used by Matlab software. Then, we further selected smaller sub-samples of 200 × 200 *μ*m (i.e., 352 × 352 pixels) from each sample, which are more common in cell mechanics [see Fig. 6(b)]. The data of each sub-sample were assembled in a 3D matrix ***A***(X = 352, Y = 352, and Z = N) where N is the number of all cross sections in the Z-stack. The total amount of color intensity for each color for all the pixels of all the cross sections of the selected sub-sample will be

$$M_{\mathrm{color}}=\sum_{i,j,k}A\left(X_{i},Y_{j},Z_{k}\right)\;\left(i,j=1,352;\;\;k=1,N\right).$$ (1)

Note that *M_color_* is a dimensionless number representing the sum of color intensity from all the cross sections throughout the co-culture thickness [shown in Fig. 2(c)].

Similarly, one can compute the color intensity of the Z-stack cross sections (i.e., slices) within each layer of the EECs and MSMCs of the uterine wall co-culture model. For this analysis, the relevant cross sections of the EECs and MSMCs layers were selected manually, and Eq. (1) was computed for the range *k* of the selected slices. The borders of each layer between the substrate and the apical end of the EEC were selected with a resolution between 1 and 2 cross sections out of 40–70. An example for the amount of F-actin filaments (i.e., green) vs Z-axis in a specific sub-sample is shown in Fig. S5(c) in the supplemental material for the complete co-culture and the EECs and MSMCs layers.

The relative alteration of the mass of a specific molecule (e.g., actin filaments) due to PWSSs (as shown in Figs. 7 and 8) was computed with respect to the non-exposed control wells in a specific experiment. The total intensity within the cross sections of either the EECs or the MSMCs ($\;M_{\mathrm{color}/\mathrm{relative}}^{\mathrm{cell}}$) of the sub-samples of exposed to PWSS was scaled by the averaged total intensity of the cross sections of either the EECs or MSMCs from all the control sub-samples (i.e., 10–15 per well). Hence,

$$M_{\mathrm{color}/\mathrm{relative}}^{\mathrm{cell}}=\frac{\sum_{i,j,k}A_{\mathrm{color}}^{\mathrm{cell}}\left(X_{i},Y_{j},Z_{k}\right)}{\frac{\sum_{q}\sum_{i,j,k}A_{{\text{control}}_{q}}^{\mathrm{cell}}\left(X_{i},Y_{j},Z_{k}\right)}{Q}}\left(i,j=1,352;\;\;k=1,N_{\mathrm{cell}};\;\;q=1,Q\right),$$ (2)

where the superscript “cell” stands for either the EECs or the MSMCs. N_cell_ is the number of cross sections in the layer of either EECs or MSMCs, and Q is the total number of the control sub-samples per test. This analysis is useful for evaluation of the relative amount of polymerization or de-polymerization of the actin or tubulin filaments in response to a biochemical or biophysical provocation.

### Statistical analysis

The relative mass of F-actin or β-tubulin was measured from the fluorescent intensity of the relevant color in the confocal images. The intensity of each experiment was scaled with respect to that of the unstressed control wells. The results are presented as $\mathrm{mean}\pm \frac{SD}{\sqrt{N}}$. Each flow experiment included several separate wells, and each well was sampled in different areas during the confocal microscopy analysis, resulting in a large number of data points. Statistical analyses of the t-test, nested t-test, and ANOVA were performed using Microsoft Excel and Matlab Software. The statistical significance was determined using one-way analysis of variance (ANOVA) followed by Tukey's HSD (honestly the significant difference) test multiple comparison tests. P-values smaller than 0.05 denoted statistically significant differences.

## SUPPLEMENTARY MATERIAL

See the supplementary material for (1) design details of the flow chamber (Figs. S1 and S2), (2) details of the driving mechanism to induce peristaltic fluid flow (Figs. S3 and S4), and (3) details of the analysis of the relative amount of cytoskeletal F-actin and β-tubulin (Fig. S5) and also for two video clips demonstrating the experimental setup in motion (Videos 1 and 2).
